# How loneliness impacts depression among Chinese college students throughout COVID-19: mediators of death anxiety and negative affect

**DOI:** 10.3389/fpsyt.2025.1512074

**Published:** 2025-10-06

**Authors:** Fangyan Lv, Run Feng, Jingbin Tan, Jie Li, Yanping Liu, Dingguo Gao

**Affiliations:** ^1^ School of Marxism, Sun Yat-Sen University, Guangzhou, China; ^2^ Guangdong Provincial Key Laboratory of Social Cognitive Neuroscience and Mental Health, Department of Psychology, Sun Yat-Sen University, Guangzhou, China

**Keywords:** loneliness, depression, death anxiety, negative affect, college students

## Abstract

**Background:**

The COVID-19 pandemic, which is considered a public crisis, has profoundly affected the psychological well-being, behavioral patterns, and daily routines of individuals across the globe. Throughout the pandemic, the specter of death anxiety, triggered by the virus, has been an ever-present shadow, constantly haunting the minds of people and causing a significant impact on their mental health. Previous studies have indicated that the practice of home isolation during the pandemic led to a substantial rise in loneliness, especially among the student population, potentially precipitating depressive emotions during COVID-19. The present study aimed to investigate the influence of loneliness on depressive symptoms in Chinese college students during the COVID-19 pandemic with two mediators: death anxiety and negative affect.

**Methods:**

This study employed a cross-sectional online design, collecting data from Chinese university students in March 2020. All participants (N=646; age M=19.960, SD=1.801; 49.690% males and 50.310% females) completed the UCLA Loneliness Scale, Self-rating Depression Scale, Templer Death Anxiety Scale in COVID-19 Context, and Negative Affect Scale. Multiple mediation analysis was utilized to analyze the data.

**Results:**

The results of this research revealed two vital findings. First, loneliness was positively correlated with death anxiety (*r* =.212, *p* <.001), negative affect (*r* =.317, *p* <.001), and depression (*r*=.545, *p* <.001). The chain mediation model showed that the risk factors of death anxiety and negative affect act as mediators in the link between loneliness and depression. This suggests that college students with higher levels of loneliness experienced increased death anxiety and negative affect, which subsequently increased depression.

**Conclusion:**

Our research offers valuable insights into the link between loneliness and depression throughout COVID-19. The findings not only enrich the empirical literature on mental health in the context of pandemics—by revealing the serial mediating role of death anxiety and negative affect—but also provide practical implications for targeted mental health interventions.

## 1 Introduction

Regarding psychological health, the COVID-19 pandemic, considered a public health crisis, has led to the highest levels of stress and anxiety among people (1, 2). During COVID-19, new measures such as quarantine affected individuals’ daily routines and overall well-being, potentially causing increased feelings of loneliness, anxiety, and depression, as well as higher rates of insomnia, substance abuse, and self-harm or suicidal tendencies (3). As an unprecedented global crisis, it has profoundly affected people’s daily lives worldwide, not only through the threat of COVID-19 death but also through a significant rise in mental health problems observed in China and globally, as documented in various studies (4–6). Similarly, this public health crisis has inevitably had a serious impact on individuals’ psychological health, resulting in stress symptoms, fear of death, and depression (7–11), among medical staff (12), and university students (13, 14). During the pandemic, loneliness was widespread among isolated people (15) and particularly common among college students (16, 17). Research indicates that chronic or severe loneliness can easily lead to psychological and behavioral problems or worsen mental health (18–20).

Loneliness is correlated to a range of negative psychological and physical issues (21). Among loneliness-related factors, depression is the most closely influential (22). So far, an inconsistent conclusion has been drawn on how loneliness leads to depression. Some studies have indicated that causality between the two might be mutual (23), but several studies revealed loneliness was a significant outcome of depressive symptoms (24). However, this research preferred the speculation that loneliness is a substantial precursor to depression. Longitudinal research showed that loneliness would predict depression (25), both at discrete points and over time (26, 27). In addition, loneliness has not only been shown to be correlated to depression (25) but also predicted increased depression over time (22). New research has uncovered that loneliness exerts a significant influence on depressive symptoms to a moderate degree (24).

Numerous investigations have examined the link between loneliness and depressive symptoms in university students (28). Experience of loneliness seemed to depend on abnormal strategies for coping with emotional problems in the younger (29), which made them more susceptible to depressive disorders (30). For instance, ruminative thought (31) and coping mechanisms (22) served as mediators in the link. Recently, the correlation between loneliness and depression has also been supported (32–34). Research has verified that college students are particularly susceptible to feelings of loneliness and implies a growing sense of isolation among university students owing to COVID-19 (17, 35, 36). A growing sense of loneliness among university students as a result of the pandemic was closely tied to depression (37, 38). Therefore, it is important to emphasize exploring loneliness, which might predict depression, and the relationship between death anxiety and depressive symptoms in Chinese college students throughout COVID-19.

Death anxiety, defined as a stable tendency to feel negative emotions from existential death concerns (39), is universal and becomes more prominent in death-related contexts (2, 40). Additionally, it impacts daily life even unconsciously (41). It emerges in childhood (42), peaks in early adulthood—with females showing higher levels than males (43)—and is more intense in younger people than the elderly, peaking in the 20s due to unfamiliarity and uncertainty about death (44–46). According to terror management theory (TMT), depressed individuals cannot buffer death anxiety (47). Loneliness may increase youth vulnerability and social detachment, fostering death anxiety (48). College students exhibit moderate death anxiety (49), and during this public health crisis, death fear rose (2), with young people’s death anxiety harming mental health and worsening existing struggles (50). Studies confirm loneliness correlates positively with death anxiety (51, 52) and predicts it in college students (53), while death anxiety links positively to depression and exacerbates it (54, 55). Pandemic quarantine, social isolation, loneliness, and death-related stimuli harmed mental health, triggering depression and death anxiety (18, 19). Thus, this study examines whether death anxiety mediates loneliness and depression in Chinese college students.

Negative affect involves a common factor in emotional distress (56), which intensifies cognitive processes, such as reflecting on adverse feelings, which often leads to the development and escalation of depression (57). Studies indicated significant positive correlations among Chinese college students’ feelings of loneliness, negative affect, and addiction to mobile phones, with negative affect fully mediating the link between loneliness and mobile phone addiction (58). In addition, individuals with high negative emotional experiences tend to be prone to pain and depression, so negative affect is considered to be linked with depression (59, 60). Vulnerability factors for psychological issues during outbreak scenarios, as previously recognized, encompass negative affect, anxiety, aversion to unforeseen events, and perceived susceptibility to illnesses (61), for example, COVID-19 (62, 63). During this public health crisis, studies indicated that negative affect was negatively associated with depression (60, 64, 65). Therefore, this research will verify whether negative affect is another mediating factor of loneliness-induced depression in university students.

Death anxiety is a universal human occurrence, yet it can be more evident when caused by health-threatening events (66). Earlier research has suggested that death anxiety is increasing generally throughout COVID-19 (67). Research has indicated that health worries due to COVID-19 may elicit fear of death (68), and people would experience various negative affect such as sadness, worry, anger, loneliness, depression, and so on throughout COVID-19 (69). A meta-analysis revealed that participants reported a relatively higher fear of death, and suggested that females have greater death anxiety and psychological distress (2). Studies indicated that death anxiety was positively associated with negative affect throughout the pandemic outbreak in China (70). Death anxiety was more common in younger than older people (71, 72). Studies on college students during this public health crisis showed it correlated with neuroticism, with perceived stress fully mediating this link (73). Thus, this study will verify whether death anxiety and negative affect could serve as sequential mediators in the link between loneliness and depression for Chinese university students throughout COVID-19.

**Figure 1:**
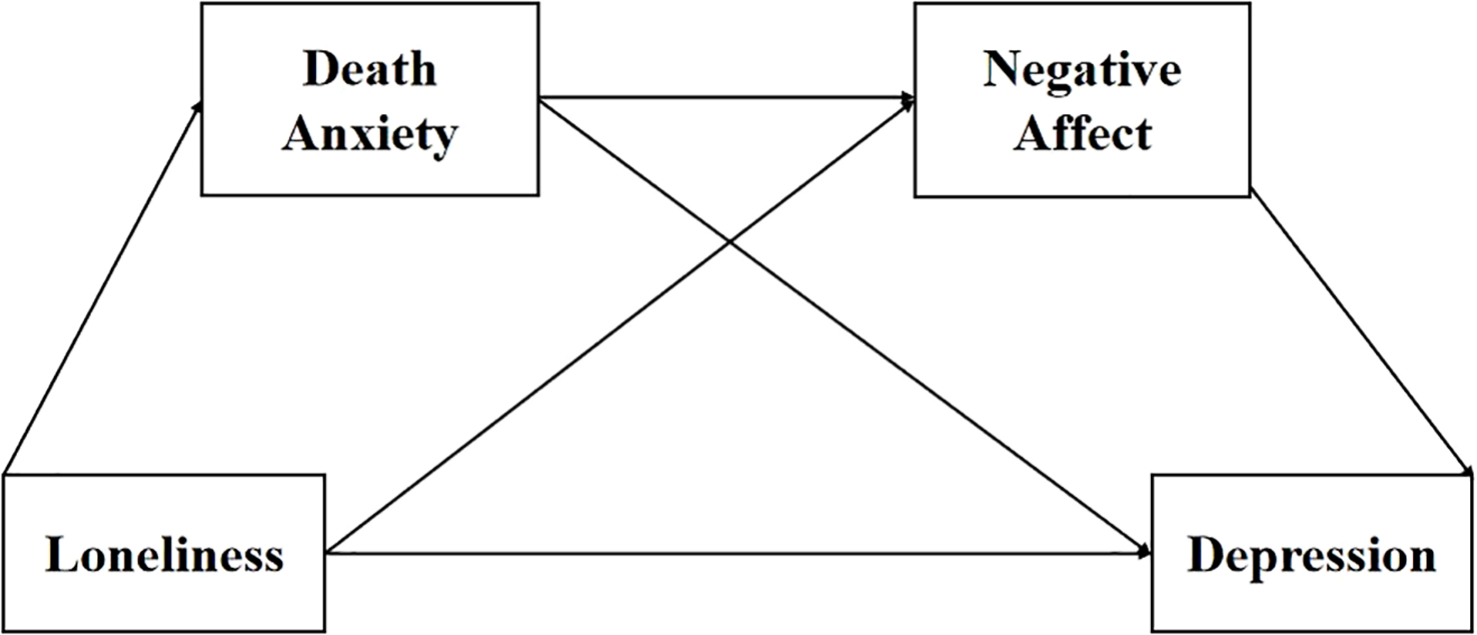
The hypothesized sequential mediation model.

To substantiate our hypotheses, this research performed an online investigation among Chinese university students to study the dynamics between loneliness and depression and the mediators of death anxiety and negative affect. We proposed that (a) there is a positive correlation between loneliness and depression in Chinese college students, (b) death anxiety acts as a mediator in the link between loneliness and depression, (c) negative affect also mediates the link between loneliness and depression, and (d) loneliness predicted college students’ depression via the sequential mediating effect of death anxiety and negative affect. We conducted a chain mediation model to evaluate the mediators of death anxiety and negative affect between loneliness and depression in Chinese university students throughout COVID-19 (see **Figure 1**).

## 2 Materials and methods

### 2.1 Participants and procedure

Initially, the sample size for this study was 655 cases. During data cleaning, 9 invalid cases were excluded in accordance with the predefined exclusion criteria. This left a final valid sample of 646 cases. These 646 cases contained no missing values and were fully incorporated into the formal statistical analysis. The time range for data collection of this study is from March 28 to May 28, 2020; meanwhile, this study is an original one. In this study, participants were recruited online from three universities of Guangdong Province in China, with males three hundred and twenty-one (49.690%) and females three hundred and twenty-five (50.310%) aged 18 years to 29 years (*M*=19.960, *SD*=1.497). All data were collected through online questionnaires on the platform Wenjuanxing (in Chinese). At the outset of the investigation, informed consent was secured. Participants were made aware that they had the freedom to exit the survey without any consequences by simply closing their browser, ensuring no response would be recorded. Questionnaire completion was self-paced. We made participants complete all measures anonymously to ensure the validity of the survey. The study design was validated and approved by the Research Ethics Committee of the author’s institution.

### 2.2 Measures

#### 2.2.1 Demographic information

In the current study, we measured demographic variables including age, gender, region (rural, urban), education (undergraduate, graduate), marital status (married, unmarried), smoking (yes, no), drinking (yes, no), and self-rated health (bad or average, good, very good), see **Table 1**.

**Table 1:** Demographic profile of the sample.

Features	Factors	n (%)	Loneliness (*M ± SD*)	Depression (*M ± SD*)
Gender	Man	321 (49.690%)	16.168 ± 4.558	34.938 ± 7.286
	Woman	325 (50.310%)	16.662 ± 4.547	35.025 ± 7.395
Age (years)	≤20	468 (72.446%)	16.406 ± 4.647	35.064 ± 7.543
	21-29	178 (27.554%)	16.444 ± 4.319	34.764 ± 6.775
Region	Urban	350(54.180%)	16.511 ± 4.512	34.803 ± 7.086
	Rural	296 (45.820%)	16.304 ± 4.612	35.193 ± 7.626
Education	Undergraduate	639 (98.916%)	16.444 ± 4.552	34.984 ± 7.345
	graduate	7 (1.084%)	13.857 ± 4.488	34.714 ± 6.945
Marital Status	Married^a^	7 (1.084%)	17.429 ± 4.315	41.000 ± 10.677
	Unmarried	639 (98.916%)	16.405 ± 4.560	34.916 ± 7.275
Smoking	Yes	25 (3.870%)	16.400 ± 4.752	39.840 ± 9.831
	No	621 (96.130%)	16.417 ± 4.552	34.786 ± 7.159
Drinking	Yes	65 (9.939%)	16.723 ± 4.665	38.785 ± 8.907
	No	581 (90.061%)	16.382 ± 4.546	34.556 ± 7.020
Self-rated health	Bad or average	60 (9.288%)	19.867 ± 4.073	43.283 ± 7.951
	Good	198 (30.650%)	17.480 ± 4.172	35.717 ± 6.938
	Very good	388 (60.062%)	15.340 ± 4.430	33.322 ± 6.467

a Including married, divorced, and widowed.

#### 2.2.2 Death anxiety

Death anxiety was evaluated using the Chinese rendition of the Templer Death Anxiety Scale (DAS, 74; CT-DAS, 75), which consists of 15 items. A 5-point scale was applied to rate each item (1= *very not agree*, 5=*very agree*). The higher scores represented worse conditions of death anxiety. Cronbach’s *α* coefficient for the CT-DAS was.91 in this measurement. The results of CFA in the present study demonstrated a good construct validity (χ^2^/df =11.492, TLI=0.897, CFI=0.918, RMSEA=0.906, SRMR=0.053).

#### 2.2.3 Negative affect

Negative affect was evaluated using the Chinese version of the Positive Affect and Negative Affect Scale (PANAS, 76). In the present research, the subscale of the Negative Affect Scale was employed to measure negative affect, including 10 items. Responses were recorded by a 5-point scale for each item (1=*never*, 5=*very strong*), with higher scores representing a higher level of negative emotion. Cronbach’s *α* coefficient for the PANAS_NA was.89. The results of CFA in the present study demonstrated a good construct validity (χ^2^/df =11.420, TLI=0.898, CFI=0.921, RMSEA=0.127, SRMR=0.039).

#### 2.2.4 Depression

The Self-rating Depression Scale (SDS, 77) is made up of 20 items by a 4-point Likert self-report questionnaire and is used to assess individuals’ depressive symptoms. Each item was evaluated using a 4-point scale (1=*never*, 4=*always*), where higher scores signified a more intense level of depressive symptoms. The Cronbach’s *α* coefficient for the SDS in the present study was.93. The results of CFA in the present study demonstrated a good construct validity (χ^2^/df =5.284, TLI=0.806, CFI=0.835, RMSEA=0.081, SRMR=0.088).

#### 2.2.5 Loneliness

The UCLA Loneliness Scale (UCLS-8, 78) is designed to assess individuals’ loneliness levels. It is a 4-point Likert self-report measurement tool that consists of eight items. A 4-point scale (1=*never*, 4=*always*) was used to rate each item, with higher scores denoting a higher intensity of loneliness. The UCLS-8 has proven to have good reliability and validity (79). The Cronbach’s *α* coefficient for the UCLS-8 in the present study was.86. The results of CFA in the present study demonstrated a good construct validity (χ^2^/df=7.806, TLI=0.897, CFI=0.926, RMSEA=0.103, SRMR=0.045).

### 2.3 Analyses

This study had no missing value data, so no data were removed. SPSS 23.0 was used for data analysis by setting a.05 (two-tailed) p-value as the criterion for statistical significance. Descriptive analyses, correlations, and mediation analyses were conducted utilizing Hayes’s PROCESS windows (Model 6) (80). Specifically, given the data’s non-normal distribution, bias-corrected confidence intervals (CI; 95%) derived from 5000 bootstrap replications were employed to assess the significance of the indirect outcome (c’ path). Confidence intervals (CI) that excluded zero would suggest significant mediation effects (80). Referring to the power tables for mediation analysis (81), estimating a notable impact (0.39) for the mediation effect (αβ), at least 71 participants were essential to attain a statistical power of at least 0.80 at the.05 significance level. Consequently, the sample size for this investigation was ample.

## 3 Results

### 3.1 Descriptive statistics

**Table 1** illustrates the demographic information gathered from every participant who participated in this online survey. From the results of correlation analysis, we observed that self-rated health was distinctly correlated to loneliness (*r* =.321, *p* <.001) and depression (*r* =.366, *p* <.001) which implies that participants with lower self-rated health might be more prone to loneliness and depression in this research, with the depression score indicating an increased risk instead of a level that is clinically significant.

As demonstrated in **Table 2**, the outcomes encompassed the Means, standard deviations, and correlation coefficients for all variables under investigation. The outcomes demonstrated that loneliness was linked positively to death anxiety, negative affect, and depressive symptoms, *r* =.212, *p* <.001, *r*=.317, *p* <.001, and *r*=.545, *p* <.001, respectively. Death anxiety was positively associated with negative affect and depression, *r* =.226, *p* <.001, and *r*=.236, *p* <.001, as well. Besides, a positive association was identified between negative affect and depression, *r*=.434, *p* <.001. The mean score of loneliness was 16.416 (SD=4.556). This mean falls approximately at the midpoint of the total score range, indicating that during this public health crisis, the participating college students generally experienced moderate levels of loneliness. Additionally, **Table 2** shows a significant positive correlation between loneliness and both death anxiety (*r*=.212, *p* <.001) and negative affect (*r*=.317, *p* <.001), confirming that moderate loneliness was accompanied by increased death anxiety and negative emotional experiences.

**Table 2:** Descriptive statistics along with the correlation outcomes of all variables.

Variables	M	*SD*	1	2	3	4	5	6
1 Loneliness	16.416	4.556	–					
2 Death anxiety	37.350	9.555	.212^***^	–				
3 Negative affect	20.786	8.026	.317^***^	.226^***^	–			
4 Depression	34.981	7.336	.545^***^	.236^***^	.434^***^	–		
5 Age	19.96	1.497	-.023	.059^**^	.052	-.003	–	
6 Gender			.054	.165^***^	-.038	.006	-.105^**^	–

*n*=646, *SD=*standard deviations. ^**^ *p* <.01, ^***^ *p* <.001.

The mean score of death anxiety was 37.350 (SD=9.555). This mean is slightly below the midpoint of the total score range (45), reflecting moderately low overall death anxiety among the sample. However, the relatively large standard deviation reveals notable individual differences: some students may have experienced heightened death anxiety due to the direct mortality threat of COVID-19 (e.g., concerns about family members’ health or their own infection risk), while others may have coped with such threats through social support or adaptive cognitive strategies (5, 13). Correlation analysis in **Table 2** further supports this interpretation: death anxiety was positively correlated with both loneliness (*r*=.212, *p* <.001) and depression (*r*=.236, *p* <.001), indicating that students with higher death anxiety tended to report greater loneliness and depressive symptoms aligning with TMD (66), which posits that mortality salience amplifies death anxiety and its links to mental health struggles.

The mean score of negative affect was 20.786 (SD=8.026). This mean is below the midpoint of the total score range (30), suggesting that the participating college students experienced moderately low overall negative affect during the study period. The SD indicates that while most students maintained relatively stable negative emotional states, a small number may have reported higher negative affect (e.g., sadness, worry, or anger) due to pandemic-related stressors (e.g., academic disruption or uncertainty about the future). As shown in **Table 2**, negative affect exhibited the strongest correlation with depression (*r*=.434, *p* <.001) among all mediators, and also correlated positively with loneliness (*r*=.317, *p* <.001) and death anxiety (*r*=.226, *p* <.001). This confirms that negative affect serves as a key “amplifier” linking loneliness and death anxiety to depression—consistent with emotion processing theories (57), which argue that unregulated negative emotions exacerbate cognitive biases and depressive symptoms.

### 3.2 Testing for multiple mediation analysis

**Figure 2:**
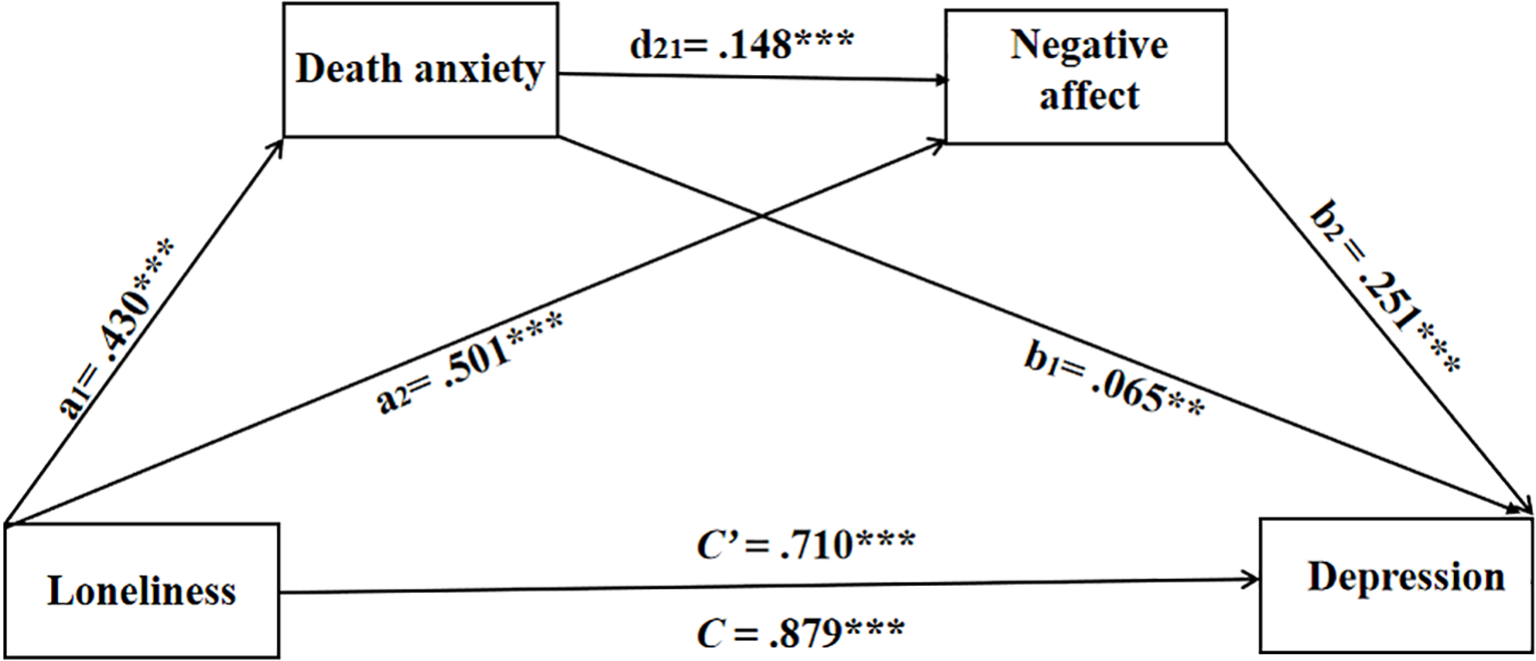
The chain mediation model. ^**^ *p* <.01, ^***^ *p* <.001.

As illustrated in **Table 3**, the chain mediation analysis was executed in the PROCESS module (Model 6) of SPSS 23.0 (the bootstrap sample size was 5000). As we hypothesized, loneliness was substantially related to depression, *b*=.879, *p* <.001, 95% *CI*=[.775,.984] (Model 1). This confirms the initial direct association between the independent variable and outcome variable, laying the foundation for subsequent mediation testing. Results showed that loneliness significantly positively predicted death anxiety, *b*=.430, *p* <.001, 95% *CI=*[.273,.586] (Model 2) (see **Figure 1** a_1_ path). This indicates that higher levels of loneliness among college students were associated with more severe death anxiety, which aligns with prior findings that pandemic-induced loneliness amplifies mortality-related worries (52, 53). Showed in Model 3, death anxiety also significantly and positively predicted negative affect, *b*=.501, *p* <.001, 95% *CI=*[.371,.631] (see **Figure 1** a_2_ path), and death anxiety also significantly and positively predicted negative affect, *b*=.148, *p* <.001, 95% *CI*=[.085,.211] (see **Figure 1** d_21_ path). These findings confirm that both loneliness and death anxiety contribute to elevated negative affect, supporting the sequential link between the two mediators (death anxiety → negative affect) in the hypothesized model. Furthermore, death anxiety (*b* =.065, *p* <.05, 95% *CI*=[.016,.115] (see **Figure 1** b_1_ path) and negative affect (*b* =.251, *p* <.001, 95% *CI*=[.191,.311] (see **Figure 1** b_2_ path) positively predicted depression, Meanwhile the impact of loneliness on depression, in a direct manner, was significant (Model 4), *b*=.710, *p*<.001, 95% *CI*=[.604,.815] (see **Figure 2** C’ path).

**Table 3:** Multiple regression of the mediation effect.

Predictors	Model1 (depression)		Model 2 (death anxiety)		Model 3 (negative affect)		Model 4 (depression)	
	*b*	*t*	*b*	*t*	*b*	*t*	*b*	*t*
Gender	-.336	-.688	3.104	4.253	-1.250^*^	-2.078	-.340	-.726
Age	.034	.209	.516	2.115	.216	1.087	-.073	-.473
Loneliness	.879^***^	16.480	.430^***^	5.391	.501^***^	7.559	.710^***^	13.237
Death anxiety					.148^***^	4.614	.065^**^	2.576
Negative affect							.251^***^	8.205
R^2^	.297		.075		.135		.379	
F	90.537^***^		17.361^***^		24.992^***^		78.133^***^	

^*^ *p* <.05, ^**^ *p* <.01, ^***^ *p* <.001.

Then, we turned to bootstrapping analyses, based on 5000 replications, to discover whether the indirect effect was substantial. The completely standardized indirect effect of loneliness on depression by way of death anxiety was substantial, *b*=.017, *p* <.05, 95% *CI*=[.004,.035]. The completely standardized indirect effect of loneliness on depression via negative affect was substantial, *b*=.078, *p* <.001, 95% *CI*=[.049,.112]. The completely standardized indirect effect of loneliness on depression via death anxiety and negative affect was substantial, *b*=.010, *p* <.001, 95% *CI*=[.004,.017]. The direct effect was *b*=.710, *p* <.001, 95% *CI*=[.604,.815]. The total indirect effect accounted for 19.226% of the total effect of loneliness on depression, confirming the validity of the sequential mediation model (see **Table 4**).

**Table 4:** Standardized indirect effects and bootstrap estimates confidence intervals.

Model pathways	Effect	Boot SE	95% Boot CI		Relative mediation effect
			LL CI	UL CI	
L→DA→D	.017^a^	.008	.074	.141	16.493%
L→NA→D	.078^a^	.016	.049	.112	74.028%
L→DA→NA→D	.010^a^	.003	.004	.017	9.479%

SE, Standard error; CI, Confidence interval; LL, Lower limit; UL, Upper limit; L, Loneliness; DA, Death anxiety; NA, Negative affect; D, Depression; ^a^Empirical 95% confidence interval excludes zero.

The findings revealed that: (1) among Chinese college students during the COVID-19 pandemic, loneliness was positively linked to depression. Loneliness showed a positive link with both death anxiety and negative affect, while depression also had a positive association with death anxiety and negative affect, and a notable correlation existed between death anxiety and negative affect. (2) The direct contribution of loneliness to university students’ depression was 68.70%. (3) The indirect contribution of loneliness to university students’ depression was 20.55%. Consequently, loneliness not only directly influenced depression but also had an indirect effect on university students’ depression through the sequential mediation of death anxiety and negative affect.

## 4 Discussion

This study examined the relationship between loneliness and depression among Chinese college students during the COVID-19 pandemic, emphasizing the sequential mediating roles of death anxiety and negative affect. The results not only confirmed the positive connection between loneliness and depression but also revealed the complex psychological process driving this link—offering new insights into pandemic-related mental health issues in young adults and informing targeted interventions. Below, we discuss these findings from theoretical, empirical, and practical viewpoints, along with limitations and future research directions.

There is a considerable risk for students to experience a boost in loneliness that correlates with depression. Our study confirms the findings from earlier studies (82, 83), and college students exhibited elevated levels of loneliness throughout COVID-19 (38). Early adulthood is a vital phase for cognitive and personality growth, and it is also the time most susceptible to mental health issues (84). Loneliness can be one of the most unpleasant experiences for college students. Evidence from across the globe suggests that college students have witnessed the most substantial spike in psychological distress rates throughout the pandemic (85).

Modeling predictors of depression established risk factors related to death anxiety and negative affect among college students. This indicated the importance of monitoring death anxiety and negative affect in youth. A study that used online ecological recognition and machine-learning predictive models to analyze posts from 17,865 active users on Weibo indicated an escalation in negative emotions (86), which is parallel to other studies (87, 88). Death anxiety, alongside the general fear of death, is a common human experience. Yet, its visibility intensifies in situations where mortality is a focal point (66), with younger individuals experiencing higher levels than their older counterparts (44, 45). The majority of the public’s reactions, both behavioral and emotional, to the virus can be comprehended through the lens of terror management theory, which posits that death anxiety is a central force influencing much of human behavior.

The study’s findings strongly align with and extend Terror Management Theory (TMT) (66), which posits that death anxiety is a core driver of psychological distress, and that social connection serves as a key “buffer” against this anxiety. Our results indicated that loneliness was positively associated with death anxiety and that death anxiety further predicted higher negative affect and depression. This supports TMT’s core tenet: when individuals lack stable social bonds, their ability to cope with mortality threats (e.g., COVID-19’s death risk) is compromised, leading to elevated death anxiety. Critically, we extended TMT by demonstrating that death anxiety does not directly drive depression in isolation—it acts as a “precursor” to negative affect, which then amplifies depressive symptoms. This sequential pathway suggests that the psychological impact of mortality salience during COVID-19 is not immediate but unfolds through cumulative emotional distress, a nuance rarely highlighted in prior TMT-based pandemic research (2, 40).

Additionally, the mediating role of negative affect aligns with emotion processing theories (57), which argue that unregulated negative emotions amplify cognitive biases (e.g., rumination about loneliness or death) and exacerbate depression. Our data showed that negative affect had the strongest correlation with depression among all mediators, and that the indirect effect of loneliness on depression via negative affect alone was far larger than the effect via death anxiety alone. This highlights that negative affect is not just a “byproduct” of loneliness or death anxiety but a central “amplifier” of depressive symptoms—consistent with studies showing that COVID-19-related negative emotions (e.g., sadness, worry) mediate mental health struggles in young adults (65, 69). By integrating TMT and emotion processing frameworks, our study offers a more holistic theoretical model for explaining how pandemic-related social isolation (loneliness) translates to depression.

Our findings are consistent with a growing body of literature on college students’ mental health during COVID-19, while also uncovering unique nuances. For instance, we confirmed prior observations that pandemic-induced loneliness correlates with higher depression in college students (37, 38, 82). Notably, our study found a strong direct correlation, which is larger than the effect sizes reported in non-pandemic studies (22, 25). This suggests that the context of COVID-19—with its mandatory home isolation, disrupted campus life, and uncertainty—may have intensified the loneliness-depression link, as social connection was actively restricted. Özgüç et al.’s (2) meta-analysis found that females report higher death anxiety during COVID-19, and our study partially supports this: gender was positively correlated with death anxiety, indicating slightly higher levels among females. However, the effect size was small, which may reflect cultural differences. Chinese college students (especially females) often receive strong family and community support during crises (5, 13), which could mitigate gender-based disparities in death anxiety—a pattern not observed in Western samples (50).

Prior research has examined either death anxiety (52, 53) or negative affect (58, 60) as single mediators in the link between loneliness and depression. Our research advances this by showing that these two factors act sequentially: loneliness first increases death anxiety (likely due to COVID-19’s mortality threat), which then heightens negative affect (as death-related worries trigger emotional distress), and finally leads to depression. The serial indirect effect may seem small, but it is theoretically critical—it reveals that ignoring either mediator would oversimplify the psychological chain linking loneliness to depression during public health crises.

## 5 Limitations

While the study provides valuable insights, it has several limitations that should be addressed in future research. First, the investigation’s main goal was to examine the elements that shape the link between loneliness and depression throughout COVID-19. The sample was limited to College students from three universities in Guangdong Province, China, limiting generalizability to students in other regions or other countries. Additionally, convenience sampling (via Wenjuanxing) may have introduced selection bias. Future studies should use stratified random sampling to include students from diverse backgrounds and regions. Second, the cross-sectional nature of the data prevents us from establishing causality. For example, while we hypothesize that loneliness predicts death anxiety and negative affect, it is also possible that depression increases loneliness (24) or that negative affect exacerbates loneliness (58). Longitudinal studies tracking students’ mental health across different phases of the pandemic would help clarify the direction of these relationships. Third, all variables were assessed via self-reported scales, which may be influenced by social desirability bias. Future research could complement self-reports with objective measures to improve data accuracy. Fourth, we did not assess factors that may moderate the observed pathways, such as social support or resilience. For example, high social support may weaken the link between loneliness and death anxiety, while resilience may buffer the effect of negative affect on depression. Including these moderators would provide a more comprehensive understanding of pandemic-related mental health. Lastly, the tested model may not provide the best fit to the data. Alternative model structures may be more parsimonious: For example, a “parallel multiple mediation model” or a “moderated mediation model” might yield better fit indices. However, our choice of sequential model was prioritized to test the theoretically derived “chain reaction” of psychological distress during COVID-19, rather than purely pursuing statistical fit. To address this limitation in future research, we propose two key adjustments: Incorporate additional variables or test alternative model structures.

## 6 Conclusion

In summary, this study demonstrates that loneliness is significantly positively associated with depression among Chinese college students during COVID-19, and this association is partially explained by the sequential mediating role of death anxiety and negative affect. The findings extend theoretical frameworks (TMT and emotion processing) to pandemic contexts, align with and refine prior empirical research, and offer actionable strategies for universities and policymakers to support students’ mental health. While limitations exist, the study highlights the importance of addressing not just direct mental health symptoms (e.g., depression) but also the underlying social and emotional factors (e.g., loneliness, death anxiety) that drive them—especially during public health crises. By targeting these interconnected factors, interventions can more effectively mitigate the long-term mental health impacts of pandemics on young adults.

## Data Availability

The raw data supporting the conclusions of this article will be made available by the authors, without undue reservation.
